# 6-Bromo-3,3-dichloro-1-methyl-1*H*-2,1-benzothia­zin-4(3*H*)-one 2,2-dioxide

**DOI:** 10.1107/S1600536809019291

**Published:** 2009-05-29

**Authors:** Muhammad Shafiq, M. Nawaz Tahir, Islam Ullah Khan, Muhammad Nadeem Arshad, Zeeshan Haider

**Affiliations:** a Government College University, Department of Chemistry, Lahore, Pakistan; b University of Sargodha, Department of Physics, Sargodha, Pakistan

## Abstract

The monomeric title compound, C_9_H_6_BrCl_2_NO_3_S, has an envelope-shaped thia­zine ring with the S atom 0.879 (9) Å out of the mean square plane of the envelope. The π–π distances between the centroids of the heterocyclic rings are 4.191 (5) and 4.110 (5) Å. The closest intermolecular inter­actions between the O atoms of the carbonyl and sulfonyl groups with Br and Cl atoms are 2.987 (7) and 2.992 (8) Å, respectively.

## Related literature

For halogination (chlorination or bromination) of 1*H*-2,1-benzothiazin-4(3*H*)-one 2,2-dioxide, see: Shafiq *et al.* (2008 ▶); Shafiq, Tahir, Khan, Ahmad *et al.* (2009 ▶); Shafiq, Tahir, Khan, Arshad & Asghar (2009 ▶); Shafiq, Tahir, Khan, Arshad & Safdar (2009 ▶).
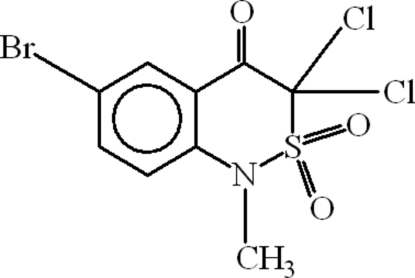


## Experimental

### 

#### Crystal data

C_9_H_6_BrCl_2_NO_3_S
*M* *_r_* = 359.02
Monoclinic, 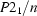

*a* = 7.0285 (9) Å
*b* = 14.865 (2) Å
*c* = 11.9739 (18) Å
β = 92.418 (5)°
*V* = 1249.9 (3) Å^3^
*Z* = 4
Mo *K*α radiation
μ = 3.88 mm^−1^
*T* = 296 K
0.26 × 0.14 × 0.12 mm

#### Data collection

Bruker Kappa-APEXII CCD diffractometer
Absorption correction: multi-scan (*SADABS*; Bruker, 2005 ▶) *T*_min_ = 0.529, *T*_max_ = 0.626
11409 measured reflections
2317 independent reflections
1595 reflections with *I* > 2σ(*I*)
*R*_int_ = 0.048

#### Refinement

*R*[*F* ^2^ > 2σ(*F* ^2^)] = 0.082
*wR*(*F* ^2^) = 0.249
*S* = 1.09
2317 reflections
155 parameters
H-atom parameters constrained
Δρ_max_ = 2.07 e Å^−3^
Δρ_min_ = −0.46 e Å^−3^

### 

Data collection: *APEX2* (Bruker, 2007 ▶); cell refinement: *SAINT* (Bruker, 2007 ▶); data reduction: *SAINT*; program(s) used to solve structure: *SHELXS97* (Sheldrick, 2008 ▶); program(s) used to refine structure: *SHELXL97* (Sheldrick, 2008 ▶); molecular graphics: *ORTEP-3 for Windows* (Farrugia, 1997 ▶) and *PLATON* (Spek, 2009 ▶); software used to prepare material for publication: *WinGX* (Farrugia, 1999 ▶) and *PLATON*.

## Supplementary Material

Crystal structure: contains datablocks global, I. DOI: 10.1107/S1600536809019291/ng2585sup1.cif

Structure factors: contains datablocks I. DOI: 10.1107/S1600536809019291/ng2585Isup2.hkl

Additional supplementary materials:  crystallographic information; 3D view; checkCIF report

## Figures and Tables

**Table 1:** Hydrogen-bond geometry (Å, °)

*D*—H⋯*A*	*D*—H	H⋯*A*	*D*⋯*A*	*D*—H⋯*A*
C7—H7*B*⋯O2	0.9600	2.3400	2.834 (14)	112.00

**Fig. 1.**
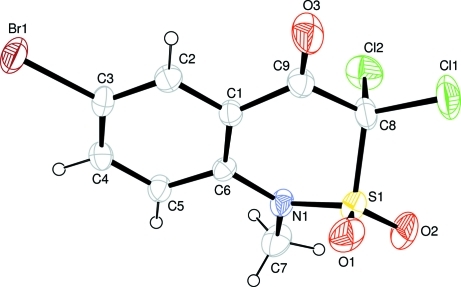
View of the title compound with the atom numbering scheme. The thermal ellipsoids are drawn at the 30% probability level. H-atoms are shown by small circles of arbitrary radii.

**Fig. 2.**
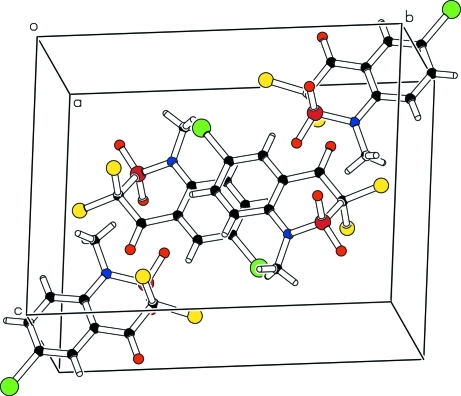
The partial packing (PLATON; Spek, 2009) which shows that molecules are stacked up with π–π interaction between the heterocyclic rings.

## supplementary crystallographic information

### Comment

We have reported crystal structures of the synthesized derivatives of the benzothiazine molecules which have halogen substitutions (Shafiq *et al.*, 2008; Shafiq, Tahir, Khan Ahmad *et al.*, 2009; Shafiq, Tahir, Khan, Arshad & Asghar, 2009; Shafiq, Tahir, Khan, Arshad & Safdar, 2009). In continuation to the halogenation of our synthesized benzothiazines, we herein report the title compound (I), (Fig. 1).

(I) is closely related to the crystal structure of 3,3-Dichloro-1-ethyl-1*H*-2,1-benzothiazin-4(3*H*)-one 2,2-dioxide, (II), (Shafiq, Tahir, Khan, Ahmad *et al.*, 2009). (I) differs from (II) due to methyl moiety at N-atom instead of ethyl and the attachement of Br-atom to the benzene ring. The title compound is from one of those compounds which have high order of steric hinderences. This is why, the R-values remain higher.

In (I), the heterocyclic ring A (C1/C6/N1/S1/C8/C9) is in the twisted form, with the maximum puckering amplitude Q_T_ = 0.604 (7) Å (Cremer & Pople, 1975). The molecules are stabilized due to weak intramolecular H-bonding (Table 1) and π—π interactions between the centroids (CgA) of ring A. The distance between CgA···CgA^i^ [symmetry code: i = 1 - *x*, 1 - *y*, 1 - *z*] is 4.191 (5) Å, whereas it is 4.110 (5) Å for CgA···CgA^ii^ [symmetry code: i = 1 - *x*, 1 - *y*, 1 - *z*]. The stacking of molecules is shown in Fig 2. The Br-atom is at a distance of -0.07 (1) Å from the mean square plane of benzene ring B (C1—C6) and the S-atom is at a distance of -0.879 (9) Å from the mean square plane of group C (C1/C6/N1/C8/C9).

### Experimental

The title compound was prepared following the method as reported in Shafiq, Tahir, Khan, Ahmad *et al.* (2009). A mixture was prepared from 6-bromo-1-methyl-1 *H*-2,1-benzothiazin-4(3*H*)-one 2,2-dioxide (250 mg, 0.862 mmol) (Shafiq, Tahir, Khan, Arshad & Asghar, 2009), N-Chloro Succinamide (225.85 mg, 1.724 mmol) and Benzoylperoxide (11.99 mg, 0.0495 mmol) in Carbon Tetra Chloride (10 ml). The mixture was heated under reflux (353 K) for two h. CCl_4_ was evaporated under reduced pressure and the residue obtained was recrystallized from mixture of ethanol:ethyl acetate (1:1).

### Refinement

H-atoms were positioned geometrically, with C—H = 0.93 and 0.96 Å for aryl and methyl H, respectively and constrained to ride on their parent atoms, with U_iso_(H) = xU_eq_(C), where x = 1.2 for aryl and 1.5 for methyl H atoms.

In difference Fourier map, three peaks of electron density 2.07, 1.87 and 1.86 e Å^-3^ exist which are at distance of 1.09, 1.09 and 1.42 Å from the BR1, BR1 and CL2, respectively.

### Crystal data

**Table:**

C_9_H_6_BrCl_2_NO_3_S	*F*(000) = 704
*M**_r_* = 359.02	*D*_x_ = 1.908 Mg m^−^^3^
Monoclinic, *P*2_1_/*n*	Mo *K*α radiation, λ = 0.71073 Å
Hall symbol: -P 2yn	Cell parameters from 2317 reflections
*a* = 7.0285 (9) Å	θ = 2.7–25.5°
*b* = 14.865 (2) Å	µ = 3.88 mm^−^^1^
*c* = 11.9739 (18) Å	*T* = 296 K
β = 92.418 (5)°	Needle, yellow
*V* = 1249.9 (3) Å^3^	0.26 × 0.14 × 0.12 mm
*Z* = 4	

### Data collection

**Table:**

Bruker Kappa-APEXII CCD diffractometer	2317 independent reflections
Radiation source: fine-focus sealed tube	1595 reflections with *I* > 2σ(*I*)
graphite	*R*_int_ = 0.048
Detector resolution: 7.80 pixels mm^-1^	θ_max_ = 25.5°, θ_min_ = 2.7°
ω scans	*h* = −8→8
Absorption correction: multi-scan (*SADABS*; Bruker, 2005)	*k* = −17→18
*T*_min_ = 0.529, *T*_max_ = 0.626	*l* = −14→14
11409 measured reflections	

### Refinement

**Table:**

Refinement on *F*^2^	Primary atom site location: structure-invariant direct methods
Least-squares matrix: full	Secondary atom site location: difference Fourier map
*R*[*F*^2^ > 2σ(*F*^2^)] = 0.082	Hydrogen site location: inferred from neighbouring sites
*wR*(*F*^2^) = 0.249	H-atom parameters constrained
*S* = 1.09	*w* = 1/[σ^2^(*F*_o_^2^) + (0.1412*P*)^2^ + 3.7083*P*] where *P* = (*F*_o_^2^ + 2*F*_c_^2^)/3
2317 reflections	(Δ/σ)_max_ < 0.000
155 parameters	Δρ_max_ = 2.07 e Å^−^^3^
0 restraints	Δρ_min_ = −0.45 e Å^−^^3^

### Special details

**Table:**

Geometry. Bond distances, angles etc. have been calculated using the rounded fractional coordinates. All su's are estimated from the variances of the (full) variance-covariance matrix. The cell esds are taken into account in the estimation of distances, angles and torsion angles
Refinement. Refinement of *F*^2^ against ALL reflections. The weighted *R*-factor *wR* and goodness of fit *S* are based on *F*^2^, conventional *R*-factors *R* are based on *F*, with *F* set to zero for negative *F*^2^. The threshold expression of *F*^2^ > σ(*F*^2^) is used only for calculating *R*-factors(gt) *etc*. and is not relevant to the choice of reflections for refinement. *R*-factors based on *F*^2^ are statistically about twice as large as those based on *F*, and *R*- factors based on ALL data will be even larger.

### Fractional atomic coordinates and isotropic or equivalent isotropic displacement parameters (Å^2^)

**Table:**

	*x*	*y*	*z*	*U*_iso_*/*U*_eq_	
Br1	0.74792 (15)	0.56286 (7)	0.70311 (9)	0.0672 (4)	
Cl1	0.7616 (5)	0.06220 (15)	0.4704 (3)	0.0880 (13)	
Cl2	0.4591 (4)	0.1801 (2)	0.3853 (3)	0.0812 (11)	
S1	0.8566 (4)	0.20955 (15)	0.3260 (2)	0.0605 (9)	
O1	1.0377 (10)	0.2088 (5)	0.3836 (7)	0.073 (3)	
O2	0.8231 (13)	0.1591 (5)	0.2266 (7)	0.092 (3)	
O3	0.7221 (14)	0.2086 (5)	0.6253 (6)	0.086 (3)	
N1	0.7709 (10)	0.3103 (5)	0.3049 (5)	0.047 (3)	
C1	0.7428 (11)	0.3339 (5)	0.5055 (7)	0.041 (2)	
C2	0.7384 (11)	0.3928 (6)	0.5972 (7)	0.046 (3)	
C3	0.7472 (11)	0.4837 (5)	0.5800 (7)	0.045 (3)	
C4	0.7598 (12)	0.5177 (5)	0.4732 (8)	0.050 (3)	
C5	0.7673 (12)	0.4611 (5)	0.3832 (7)	0.046 (3)	
C6	0.7602 (10)	0.3681 (5)	0.3979 (6)	0.037 (2)	
C7	0.7331 (16)	0.3430 (8)	0.1909 (7)	0.069 (4)	
C8	0.7023 (14)	0.1722 (5)	0.4321 (8)	0.055 (3)	
C9	0.7248 (12)	0.2370 (6)	0.5326 (7)	0.048 (3)	
H2	0.72951	0.37007	0.66913	0.0547*	
H4	0.76319	0.57957	0.46234	0.0596*	
H5	0.77715	0.48484	0.31182	0.0551*	
H7A	0.83703	0.38008	0.16917	0.1043*	
H7B	0.71969	0.29273	0.14086	0.1043*	
H7C	0.61767	0.37762	0.18786	0.1043*	

### Atomic displacement parameters (Å^2^)

**Table:**

	*U*^11^	*U*^22^	*U*^33^	*U*^12^	*U*^13^	*U*^23^
Br1	0.0732 (8)	0.0623 (7)	0.0664 (7)	−0.0014 (4)	0.0073 (5)	−0.0331 (5)
Cl1	0.140 (3)	0.0300 (12)	0.094 (2)	0.0046 (13)	0.006 (2)	0.0024 (12)
Cl2	0.0585 (16)	0.098 (2)	0.086 (2)	−0.0217 (14)	−0.0094 (14)	−0.0057 (16)
S1	0.0730 (18)	0.0455 (13)	0.0641 (15)	0.0053 (11)	0.0174 (13)	−0.0116 (11)
O1	0.047 (4)	0.076 (5)	0.096 (5)	0.017 (3)	0.005 (4)	−0.008 (4)
O2	0.136 (7)	0.063 (5)	0.078 (5)	−0.002 (5)	0.028 (5)	−0.035 (4)
O3	0.164 (8)	0.048 (4)	0.046 (4)	0.001 (4)	0.006 (4)	0.012 (3)
N1	0.075 (5)	0.036 (4)	0.031 (4)	0.003 (3)	0.005 (3)	−0.002 (3)
C1	0.045 (4)	0.034 (4)	0.043 (4)	0.000 (3)	0.002 (3)	−0.001 (3)
C2	0.056 (5)	0.048 (5)	0.033 (4)	−0.002 (4)	0.001 (4)	−0.001 (4)
C3	0.051 (5)	0.034 (4)	0.049 (5)	0.000 (3)	−0.003 (4)	−0.014 (4)
C4	0.057 (5)	0.031 (4)	0.061 (6)	0.003 (4)	0.008 (4)	0.003 (4)
C5	0.068 (6)	0.034 (4)	0.037 (4)	0.001 (4)	0.005 (4)	0.001 (3)
C6	0.041 (4)	0.042 (4)	0.029 (4)	0.003 (3)	0.002 (3)	−0.001 (3)
C7	0.102 (8)	0.073 (7)	0.033 (5)	0.014 (6)	0.002 (5)	−0.001 (4)
C8	0.082 (7)	0.028 (4)	0.054 (5)	−0.006 (4)	0.002 (5)	−0.001 (4)
C9	0.059 (5)	0.041 (4)	0.044 (5)	0.000 (4)	−0.001 (4)	0.003 (4)

### Geometric parameters (Å, °)

**Table:**

Br1—C3	1.886 (8)	C1—C9	1.483 (12)
Cl1—C8	1.744 (8)	C2—C3	1.369 (12)
Cl2—C8	1.780 (10)	C3—C4	1.381 (12)
S1—O1	1.422 (8)	C4—C5	1.370 (12)
S1—O2	1.418 (8)	C5—C6	1.395 (11)
S1—N1	1.630 (8)	C8—C9	1.544 (12)
S1—C8	1.793 (10)	C2—H2	0.9300
O3—C9	1.189 (11)	C4—H4	0.9300
N1—C6	1.411 (10)	C5—H5	0.9300
N1—C7	1.463 (11)	C7—H7A	0.9600
C1—C2	1.406 (12)	C7—H7B	0.9600
C1—C6	1.395 (11)	C7—H7C	0.9600
			
Br1···O3^i^	2.987 (7)	C3···C4^ii^	3.593 (12)
Br1···C5^ii^	3.741 (9)	C3···C4^iii^	3.550 (12)
Br1···C5^iii^	3.620 (9)	C3···C5^iii^	3.519 (11)
Br1···H7C^ii^	3.0600	C4···O2^ix^	3.219 (12)
Cl1···O1	3.125 (8)	C4···C3^ii^	3.593 (12)
Cl1···O2	3.300 (9)	C4···C3^iii^	3.550 (12)
Cl1···O3	2.880 (8)	C4···C4^iii^	3.451 (12)
Cl2···O1^iv^	2.992 (8)	C5···Br1^ii^	3.741 (9)
Cl2···O2	3.266 (9)	C5···Br1^iii^	3.620 (9)
Cl2···O3	3.377 (9)	C5···O2^ix^	3.275 (11)
Cl2···N1	3.107 (8)	C5···C3^iii^	3.519 (11)
Cl2···C6	3.505 (8)	C6···Cl2	3.505 (8)
Cl2···H2^v^	3.0800	C5···H7C	2.8100
O1···Cl1	3.125 (8)	C5···H7A	2.8900
O1···Cl2^vi^	2.992 (8)	C7···H5	2.5700
O1···C1	3.184 (11)	H2···O3	2.4600
O2···Cl1	3.300 (9)	H2···Cl2^x^	3.0800
O2···Cl2	3.266 (9)	H4···O2^ix^	2.6000
O2···C4^vii^	3.219 (12)	H5···C7	2.5700
O2···C5^vii^	3.275 (11)	H5···H7A	2.3600
O3···Cl2	3.377 (9)	H5···H7C	2.4200
O3···Cl1	2.880 (8)	H5···O2^ix^	2.7200
O3···Br1^viii^	2.987 (7)	H7A···C5	2.8900
O2···H7B	2.3400	H7A···H5	2.3600
O2···H5^vii^	2.7200	H7B···O2	2.3400
O2···H4^vii^	2.6000	H7C···C5	2.8100
O3···H2	2.4600	H7C···H5	2.4200
N1···Cl2	3.107 (8)	H7C···Br1^ii^	3.0600
C1···O1	3.184 (11)		
			
O1—S1—O2	121.1 (5)	Cl1—C8—Cl2	111.1 (5)
O1—S1—N1	113.6 (4)	Cl1—C8—S1	109.4 (5)
O1—S1—C8	102.1 (5)	Cl1—C8—C9	111.4 (6)
O2—S1—N1	108.0 (4)	Cl2—C8—S1	111.0 (5)
O2—S1—C8	110.3 (5)	Cl2—C8—C9	105.6 (6)
N1—S1—C8	99.4 (4)	S1—C8—C9	108.3 (6)
S1—N1—C6	118.0 (5)	O3—C9—C1	123.7 (8)
S1—N1—C7	120.1 (6)	O3—C9—C8	120.1 (8)
C6—N1—C7	121.4 (7)	C1—C9—C8	116.2 (7)
C2—C1—C6	120.0 (7)	C1—C2—H2	120.00
C2—C1—C9	115.4 (7)	C3—C2—H2	120.00
C6—C1—C9	124.6 (7)	C3—C4—H4	120.00
C1—C2—C3	119.7 (8)	C5—C4—H4	120.00
Br1—C3—C2	119.8 (6)	C4—C5—H5	120.00
Br1—C3—C4	119.8 (6)	C6—C5—H5	120.00
C2—C3—C4	120.4 (8)	N1—C7—H7A	109.00
C3—C4—C5	120.6 (7)	N1—C7—H7B	109.00
C4—C5—C6	120.4 (7)	N1—C7—H7C	109.00
N1—C6—C1	121.1 (7)	H7A—C7—H7B	109.00
N1—C6—C5	120.0 (7)	H7A—C7—H7C	109.00
C1—C6—C5	118.9 (7)	H7B—C7—H7C	109.00
			
O1—S1—N1—C6	−52.8 (7)	C2—C1—C6—N1	178.0 (7)
O1—S1—N1—C7	118.5 (8)	C2—C1—C6—C5	−1.9 (11)
O2—S1—N1—C6	169.9 (6)	C9—C1—C6—N1	−3.0 (12)
O2—S1—N1—C7	−18.7 (9)	C9—C1—C6—C5	177.1 (8)
C8—S1—N1—C6	54.9 (7)	C2—C1—C9—O3	−5.9 (13)
C8—S1—N1—C7	−133.8 (7)	C2—C1—C9—C8	172.1 (7)
O1—S1—C8—Cl1	−64.1 (6)	C6—C1—C9—O3	175.1 (9)
O1—S1—C8—Cl2	173.0 (5)	C6—C1—C9—C8	−6.9 (12)
O1—S1—C8—C9	57.5 (7)	C1—C2—C3—Br1	−178.4 (6)
O2—S1—C8—Cl1	65.9 (6)	C1—C2—C3—C4	0.2 (12)
O2—S1—C8—Cl2	−57.0 (6)	Br1—C3—C4—C5	177.3 (6)
O2—S1—C8—C9	−172.5 (6)	C2—C3—C4—C5	−1.2 (12)
N1—S1—C8—Cl1	179.2 (5)	C3—C4—C5—C6	0.7 (13)
N1—S1—C8—Cl2	56.3 (5)	C4—C5—C6—N1	−179.1 (7)
N1—S1—C8—C9	−59.3 (6)	C4—C5—C6—C1	0.8 (12)
S1—N1—C6—C1	−27.0 (10)	Cl1—C8—C9—O3	−21.8 (12)
S1—N1—C6—C5	152.9 (6)	Cl1—C8—C9—C1	160.1 (6)
C7—N1—C6—C1	161.8 (8)	Cl2—C8—C9—O3	98.9 (9)
C7—N1—C6—C5	−18.3 (11)	Cl2—C8—C9—C1	−79.2 (8)
C6—C1—C2—C3	1.4 (12)	S1—C8—C9—O3	−142.1 (8)
C9—C1—C2—C3	−177.7 (7)	S1—C8—C9—C1	39.8 (9)

Symmetry codes: (i) −*x*+3/2, *y*+1/2, −*z*+3/2; (ii) −*x*+1, −*y*+1, −*z*+1; (iii) −*x*+2, −*y*+1, −*z*+1; (iv) *x*−1, *y*, *z*; (v) *x*−1/2, −*y*+1/2, *z*−1/2; (vi) *x*+1, *y*, *z*; (vii) −*x*+3/2, *y*−1/2, −*z*+1/2; (viii) −*x*+3/2, *y*−1/2, −*z*+3/2; (ix) −*x*+3/2, *y*+1/2, −*z*+1/2; (x) *x*+1/2, −*y*+1/2, *z*+1/2.

### Hydrogen-bond geometry (Å, °)

**Table:**

*D*—H···*A*	*D*—H	H···*A*	*D*···*A*	*D*—H···*A*
C7—H7B···O2	0.9600	2.3400	2.834 (14)	112.00
